# Supplementary material for: Low thoracic skeletal mass index, a novel marker to predict recurrence of aspiration pneumonia in the elderly stroke patients
Source: PLoS One. 2024 Dec 12;19(12):e0315427. doi: 10.1371/journal.pone.0315427 (PMC11637400; doi:10.1371/journal.pone.0315427)

**Suppl** Comparison of time to recurrence (days) of aspiration pneumonia in patients with dysphagia according to T2MI quartiles


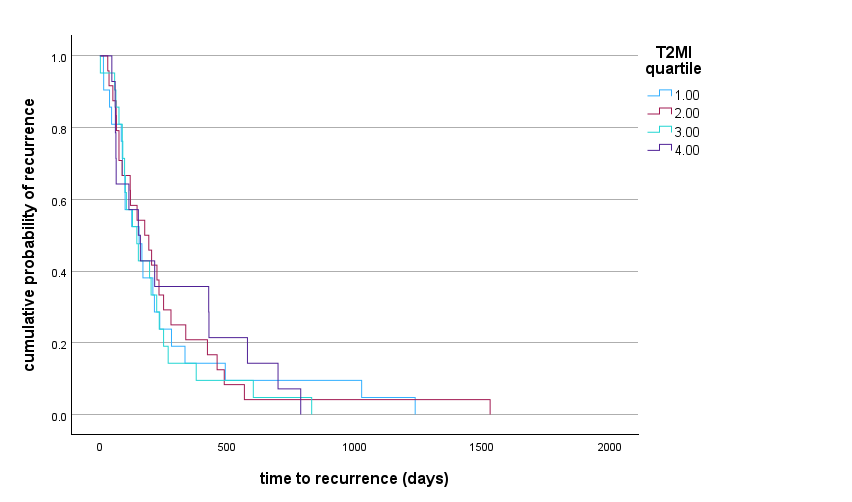

Supplement: S1 Fig — (DOCX) [file pone.0315427.s001.docx]
