# Supplementary material for: Low thoracic skeletal mass index, a novel marker to predict recurrence of aspiration pneumonia in the elderly stroke patients
Source: PLoS One. 2024 Dec 12;19(12):e0315427. doi: 10.1371/journal.pone.0315427 (PMC11637400; doi:10.1371/journal.pone.0315427)
Supplement: Attachment — Submitted filename: response to reviewers.docx [file pone.0315427.s003.docx]

**Dear Editor-in-Chief,**

We would like to thank you and the reviewers of the *PLOS ONE* for taking the time to review our article. It is truly honorable to receive the letter of revision to enrich the work of research we’ve performed. We’d like to appreciate for the time and efforts by the editors to this paper. We have made some corrections and clarifications in the manuscript after going over the reviewers’ comments.

The changes are summarized below:

Journal requirements:

When submitting your revision, we need you to address these additional requirements.

1. Please ensure that your manuscript meets PLOS ONE's style requirements, including those for file naming. The PLOS ONE style templates can be found at

https://journals.plos.org/plosone/s/file?id=wjVg/PLOSOne_formatting_sample_main_body.pdf and

A clean copy of the edited manuscript (uploaded as the new *manuscript* file)

3. Note from Emily Chenette, Editor in Chief of PLOS ONE, and Iain Hrynaszkiewicz, Director of Open Research Solutions at PLOS: Did you know that depositing data in a repository is associated with up to a 25% citation advantage (https://doi.org/10.1371/journal.pone.0230416)? If you’ve not already done so, consider depositing your raw data in a repository to ensure your work is read, appreciated and cited by the largest possible audience. You’ll also earn an Accessible Data icon on your published paper if you deposit your data in any participating repository (https://plos.org/open-science/open-data/#accessible-data).

4. Thank you for stating the following financial disclosure:

“This work was supported by a National Research Foundation of Korea (NRF) grant funded by the Korean government (no. 2020R1F1A1065814) and Technology Innovation Program (No. 20018182) funded by the Ministry of Trade, Industry & Energy (MOTIE, Korea),

Please state what role the funders took in the study.  If the funders had no role, please state: "The funders had no role in study design, data collection and analysis, decision to publish, or preparation of the manuscript."

If this statement is not correct you must amend it as needed.

Please include this amended Role of Funder statement in your cover letter; we will change the online submission form on your behalf.

: As the editor suggested, we added the sentences about the role of funders, and we also additional financial disclosure additionally.

5. We note that you have indicated that there are restrictions to data sharing for this study. PLOS only allows data to be available upon request if there are legal or ethical restrictions on sharing data publicly. For more information on unacceptable data access restrictions, please see http://journals.plos.org/plosone/s/data-availability#loc-unacceptable-data-access-restrictions.

Before we proceed with your manuscript, please address the following prompts:

: We allow data to be available upon request.

a) If there are ethical or legal restrictions on sharing a de-identified data set, please explain them in detail (e.g., data contain potentially identifying or sensitive patient information, data are owned by a third-party organization, etc.) and who has imposed them (e.g., a Research Ethics Committee or Institutional Review Board, etc.). Please also provide contact information for a data access committee, ethics committee, or other institutional body to which data requests may be sent.

b) If there are no restrictions, please upload the minimal anonymized data set necessary to replicate your study findings to a stable, public repository and provide us with the relevant URLs, DOIs, or accession numbers. For a list of recommended repositories, please see

https://journals.plos.org/plosone/s/recommended-repositories. You also have the option of uploading the data as Supporting Information files, but we would recommend depositing data directly to a data repository if possible.

We will update your Data Availability statement on your behalf to reflect the information you provide.

: We will upload our data as supporting information files.

6. In this instance it seems there may be acceptable restrictions in place that prevent the public sharing of your minimal data. However, in line with our goal of ensuring long-term data availability to all interested researchers, PLOS’ Data Policy states that authors cannot be the sole named individuals responsible for ensuring data access (http://journals.plos.org/plosone/s/data-availability#loc-acceptable-data-sharing-methods).

Data requests to a non-author institutional point of contact, such as a data access or ethics committee, helps guarantee long term stability and availability of data. Providing interested researchers with a durable point of contact ensures data will be accessible even if an author changes email addresses, institutions, or becomes unavailable to answer requests.

Before we proceed with your manuscript, please also provide non-author contact information (phone/email/hyperlink) for a data access committee, ethics committee, or other institutional body to which data requests may be sent. If no institutional body is available to respond to requests for your minimal data, please consider if there any institutional representatives who did not collaborate in the study, and are not listed as authors on the manuscript, who would be able to hold the data and respond to external requests for data access? If so, please provide their contact information (i.e., email address). Please also provide details on how you will ensure persistent or long-term data storage and availability.

7. Please ensure that you include a title page within your main document. You should list all authors and all affiliations as per our author instructions and clearly indicate the corresponding author.

: As the editor requested, we included a title page within our main document.

Additional Editor Comments:

Please address the reviewers' comments appropriately. If not, I cannot guarantee acceptance.


[Note: HTML markup is below. Please do not edit.]

Reviewers' comments:

Reviewer's Responses to Questions

**Comments to the Author**

5. Review Comments to the Author

Please use the space provided to explain your answers to the questions above. You may also include additional comments for the author, including concerns about dual publication, research ethics, or publication ethics. (Please upload your review as an attachment if it exceeds 20,000 characters)

Reviewer #1: This is a retrospective analysis of patients diagnosed with stroke who developed aspiration pneumonia and whether thoracic skeletal muscle index was a predictor for aspiration pneumonia recurrence. The authors analyzed data from 2014-2020 in a single center. Aspiration pneumonia was defined as clinical signs/symptoms of pneumonia in the dependent portions of the lung. Measured thoracic muscle volume used the CSA of the erector spine muscle at the 12th vertebral region. Those with a lower T12MI were more likely to have a recurrence based on multivariate logistic regression analysis. Overall the paper appears to demonstrate a novel association in a large cohort of patients.

I had some questions related to their methodology. There were also a few instances in the paper where the authors made statements which may be debatable (such as "80% of pneumonias that require hospitalization are aspiration pneumonias). Would highly recommend that the authors double check that they are using citations appropriately and with the correct context.

: Thank you for your valuable pointing that out. We corrected sentences and detailed specific population. (Line 51-54)

Major criteria:
• The authors divided ESM by height to develop the thoracic skeletal muscle index. Did the authors consider dividing by BMI? Several authors have divided by body size (i.e. BMI) instead of height. Please provide the rationale for dividing by height and not BMI.

: Thank you for your valuable comments. Sarcopenia index is different from studies. In some prediction models for sarcopenia, skeletal muscle, sex, age and body weight is included ([Eur Arch Otorhinolaryngol.](https://www.ncbi.nlm.nih.gov/pmc/articles/PMC10620319/) 2023; 280(12): 5583–5594.). We are based on previous studies using ESM and patients’ height squared, and we cited references in line 176.

• In the introduction, the authors state that aspiration pneumonia is responsible for 80% of pneumonia cases that require hospitalization, however the paper that they cite demonstrated 80% for those 70 years and older. The overall prevalence of AP was 60% of hospitalized patients with CAP. Would ensure that the authors are clear what population they are referring to when using citations. Would also include other papers and include a range of incidence for aspiration pneumonia as the cause of CAP in hospitalized patients. Most citations state that 5-15% of cases of CAP are due to aspiration pneumonia, although these may include non-hospitalized patients.

: As the reviewer suggested, we corrected sentences and detailed specific population. (Line 54). And also added incidence of AP as the cause of CAP in hospitalized patients (Line 51-54)

• In your methods, please include the rationale and evidence for the the muscle density you use (40 to 100 HU). Many studies use a different range (-50 to +90 for instance). (PMID: 24558953)

: In previous study assess body composition, normal-density muscle is defined as having attenuation values in the 40–100 HU range. We cited related references in line 176.

• The authors quantified the time to recurrence of aspiration pneumonia, but I was surprised they did not do a survival analysis.

: Thank you for your valuable comments. As the reviewer suggested, we did survival analysis. T2MI is continuous variables, we grouped patients into 4 groups according to T2MI quartile, but there were no differences among 4 groups in Kaplan Meier survival analysis (Log rank 0.916). We will upload as supplementary files.

• In the results section, the authors describe Hazard ratios but it would be helpful to provide an interpretation for the reader. Such as “each drop in T12MI by XX was associated with an increase in AP recurrence by XX”

: As the reviewer suggested, we changed the expressions to clarify interpretation for the reader (Line 242-251)

• The authors state that “For this study, we conducted a retrospective review of data for patients diagnosed with dysphagia disorder due to stroke.” -> Do we know when the stroke was diagnosed? Are these recent strokes or happened years prior?

: As the reviewer pointed out, we specified stroke period including acute and subacute stroke. In line 96-97 and 100-101)

Minor criteria:
• Methods lines 113-114, “In stroke patients with severe facial palsy and unable to perfect lip seal, the therapist aided by holding the lips around the mouthpiece minimize air leak." -> I believe the authors meant "around the mouthpiece TO minimize air leak"

: As the reviewer suggested, we added ‘to’ to clarify meaning in lines 138.

• Please double check your citations. The authors state: “In a previous study, ESMCSA assessed by chest CT was a valuable clinical parameter to correlate symptoms and disease prognosis in patients with chronic obstructive lung disease.[37] “ but the citation for 37 is a basic science paper unrelated to ESM.

: As the reviewer pointed out, we reviewed and cited other reference.

• In the discussion the authors state: “Most stroke-related pneumonia is dysphagia related AP” - please provide a citation here.

: As the reviewer suggested, we cited references additionally.

Reviewer #2:

1. This manuscript presents the potential of low thoracic muscle index as an effective predictive factor for the recurrence of aspiration pneumonia in patients with stroke. While the paper is well-written, I have some minor concerns.

2. Person-first language (e.g., patient with stroke not stroke patient; or person with diabetes not a diabetic) is highly recommended in academic writing.
<https://www.nih.gov/nih-style-guide/person-first-destigmatizing-language>

: Thank you for reviewer’s valuable comments. As the reviewer suggested, we corrected several phrases to person-first language.

3. Discussion line 249-250 “Our results showed that those at the time of AP diagnosis all patients already” The sentence appears to be incomplete.

: As the reviewer pointed out, we reviewed and deleted incomplete sentences.

4. Please check the abbreviation (e.g., confidence interval [CI] and aspiration pneumonia [AP]). To ensure clarity and readability, please use abbreviations consistently once they have been introduced.

: As the reviewer suggested, we rechecked the use of abbreviations and used consistently.

5. It would be beneficial to include some other potential confounding factors, such as the severity of stroke or the degree of dysphagia, in multivariate analysis if feasible.

: We agreed the reviewer’s opinion. However, other dysphagic parameters and markers for functional disability except PCF were not significantly different between recurrence and non-recurrence groups. So, we included only min PCF for univariate and multivariate analysis.
