# Supplementary material for: Low thoracic skeletal mass index, a novel marker to predict recurrence of aspiration pneumonia in the elderly stroke patients
Source: PLoS One. 2024 Dec 12;19(12):e0315427. doi: 10.1371/journal.pone.0315427 (PMC11637400; doi:10.1371/journal.pone.0315427)
Supplement: Attachment — Submitted filename: response to reviewers_20241114.docx [file pone.0315427.s004.docx]

**Dear Editor-in-Chief,**

We would like to thank you and the reviewers of the *PLOS ONE* for taking the time to review our article. It is truly honorable to receive the letter of revision to enrich the work of research we’ve performed. We’d like to appreciate for the time and efforts by the editors to this paper. We have made some corrections and clarifications in the manuscript after going over the reviewers’ comments.

The changes are summarized below:

Journal Requirements:

Please review your reference list to ensure that it is complete and correct. If you have cited papers that have been retracted, please include the rationale for doing so in the manuscript text, or remove these references and replace them with relevant current references. Any changes to the reference list should be mentioned in the rebuttal letter that accompanies your revised manuscript. If you need to cite a retracted article, indicate the article’s retracted status in the References list and also include a citation and full reference for the retraction notice.

: As the editor requested, we did review our reference list, and there is no changes to the reference list.

Additional Editor Comments:

This is an excellent paper. Please see my minor comments.
#1. Considering that this study is retrospective, compliance with the Declaration of Helsinki is not required.

: As the editor requested, we deleted the sentence about the Declaration of Helsinki.
#2. In line 130, PCF, Maximal expiratory pressures (MEP) -> PCF, maximal expiratory pressures (MEP)

: Thank you for careful review. We corrected Maximal as maximal.
#3. Considering that SPSS is now available up to version 29, did you indeed conduct the statistical analysis using version 20?

: We checked again, and our institution is still use SPSS version 20.
#4. In a chart review study, it is essential to describe any missing variables that may naturally arise. This aspect appears to be missing from the manuscript.

: As the editor suggested, we added sentences about the issue of missing variables in limitation (line 347-350)

#5. In Table 1, please describe SMD value instead of P value.

: We calculated standardized mean differences for continuous variables and added values at table 1.
#6. What covariates were selected for adjustment in the multivariate analysis? I find it difficult to locate this information in the manuscript. Please add it to the table footnote.

: We added variables selected for adjustment in the multivariate analysis at the table 4 footnote.
